# The genomic landscape of chronic lymphocytic leukemia: clinical implications

**DOI:** 10.1186/1741-7015-11-124

**Published:** 2013-05-09

**Authors:** Víctor Quesada, Andrew J Ramsay, David Rodríguez, Xose S Puente, Elías Campo, Carlos López-Otín

**Affiliations:** 1 Departamento de Bioquímica y Biología Molecular, Universidad de Oviedo - IUOPA, Oviedo, Spain; 2 Unidad de Hematopatología, Servicio de Anatomía Patológica, Hospital Clínic, Universitat de Barcelona, IDIBAPS, Barcelona, Spain

**Keywords:** Chronic lymphocytic leukemia, Genomics, Epigenomics, Driver mutations, Personalized medicine

## Abstract

A precise understanding of the genomic and epigenomic features of chronic lymphocytic leukemia (CLL) may benefit the study of the disease’s staging and treatment. While recent reports have shed some light on these aspects, several challenges need to be addressed before translating this research into clinical practice. Thus, even the best candidate driver genes display low mutational rates compared to other tumors. This means that a large percentage of cases do not display clear tumor-driving point mutations, or show candidate driving point mutations with no obvious biochemical relationship to the more frequently mutated genes. This genomic landscape probably reflects either an unknown underlying biochemical mechanism playing a key role in CLL or multiple biochemical pathways independently driving the development of this tumor. The elucidation of either scenario will have important consequences on the clinical management of CLL. Herein, we review the recent advances in the definition of the genomic landscape of CLL and the ongoing research to characterize the underlying biochemical events that drive this disease.

## Chronic lymphocytic leukemia

Chronic lymphocytic leukemia (CLL) is a clonal neoplasia of B-lymphocytes which accumulate mainly in the blood, bone marrow, lymph nodes and spleen [1,2]. Notably, these B-lymphocytes are differentiated, and can remain in an arrested state for several years after diagnosis. In spite of its high prevalence in Western countries, and the consequent interest on its biological and clinical features, the characterization of the genomic events that drive this disease proved cumbersome to classical molecular biology approaches. The main success in this regard was the categorization of CLL in two groups, depending on whether the tumoral B-cells had undergone the somatic hypermutation (SHM) process in the germinal center (*IGHV*-mutated) or not (*IGHV*-unmutated) [3]. While *IGHV*-mutated CLL patients frequently show mild clinical features and high overall survival (OS) and progression-free survival (PFS), *IGHV*-unmutated CLL patients suffer an aggressive form of the disease which may be refractory to treatment [4]. This broad classification system was improved with the characterization of additional genomic and transcriptomic factors, like inactivating mutations in *TP53*[5] and *ATM*[6] and expression of ZAP70 [7] and CD38 [8]. Despite the relatively stable karyotype in CLL cells, genomic aberrations were also recognized as important determinants of the clinical course of the disease [9]. Among these variants, a homozygous deletion at 13q14 which affects two micro-RNA genes (miR15-a and miR16-1) was suggested as a triggering event of CLL in about 50% of the patients [10,11]. However, the genomic events that dictate the initiation and heterogeneous evolution of CLL remained partially unknown.

## Genomic alterations in chronic lymphocytic leukemia

Next-Generation Sequencing (NGS) techniques have offered, for the first time, an essentially complete and unbiased picture of a genome in a time- and cost-effective way, thanks to their ability to read millions of DNA fragments in one experiment [12]. This, in turn, allows the comparison of the genome of tumor cells with the constitutive genome in normal tissues of the same patient. The variants present in the tumor genome and absent from the germinal genome are called somatic mutations, and constitute a requisite of cancer development [13]. With different types of input material and sample processing, these techniques can detect somatic point mutations and small insertion/deletions (whole-genome, whole-exome), chromosome rearrangements (whole-genome), expression and splicing variants (RNA-Seq) and changes in the epigenome (ChIP-Seq, bisulfite-based methods) [14,15]. Notably, whole-exome sequencing is a two-step procedure in which all known coding regions of the genome are first purified by targeted capture and then sequenced. Since these regions span less than 5% of the genome, this procedure retrieves information on protein-altering mutations for a small fraction of the cost of whole-genome sequencing.

Most of the somatic variants in cancer are passenger mutations, acquired randomly during the lifetime of the original tumor cell before or during malignant transformation, and provide no advantageous survival phenotype. By contrast, a few driver mutations contribute to the transformation process or allow tumor cells to survive pharmacological treatments [16]. The primary strategy to find driver mutations in a tumor type is inter-tumor recurrence. Because of their functional role, driver mutations are expected to affect a restricted number of tumor suppressors and oncogenes, and, therefore, somatic mutations affecting these genes will be found in multiple patients with the same type of tumor. To facilitate this strategy, the International Cancer Genome Consortium (ICGC) [17] and the Cancer Genome Atlas (TCGA) (http://cancergenome.nih.gov/) were formed to sequence the genomes, transcriptomes and epigenomes of large cohorts of paired tumor and normal samples from the most prevalent cancer types.

Given its clinical and societal impact and the gaps in our knowledge of its genomic determinants, CLL was included among the 50 tumors prioritized by the ICGC for large-scale sequencing. The first result of this effort was the sequencing and analysis of the whole CLL genome in two *IGHV*-mutated and two *IGHV*-unmutated cases [18]. This work showed for the first time the usefulness of next-generation sequencing technologies to find novel genetic drivers of CLL. Thus, the study described about 1,000 somatic variants per patient, with 45 genes affected by protein-altering somatic mutations. Only four of those genes were found to be expressed and recurrently mutated in a validation set of 169 additional samples. Therefore, these genes, *NOTCH1*, *MYD88*, *XPO1* and *KLHL6*, were singled out as drivers of CLL. Only *NOTCH1* had been previously related to CLL and other lymphoid malignancies [19]. Functional studies showed that the observed mutation, a recurrent small deletion of two coding bases, produces a truncated form of NOTCH1 that accumulates in the cell. A simultaneous study and additional subsequent studies have confirmed these findings, concluding that *NOTCH1* somatic mutation is an independent prognostic factor for aggressive forms of CLL [20-22]. Therefore, this gene provides an attractive target for pharmacological intervention [23]. In addition to the discovery of recurrent somatic point mutations, the mutational profile of the *IGHV*-mutated samples suggested that a significant fraction of the somatic variations could be caused by activation of the error-prone polymerase-η, a hallmark of the normal SHM process. This observation relates the clinical differences between the *IGHV*-mutated and *IGHV*-unmutated CLL subtypes to the underlying genomic events.

**Figure 1:**
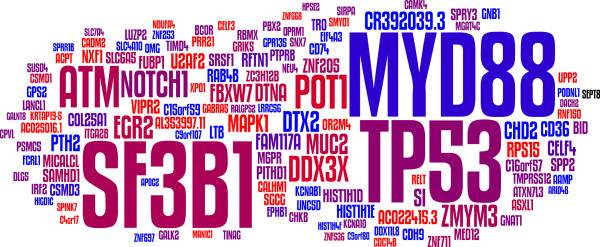
**Recurrent mutations in CLL.** The size of each gene symbol is proportional to the logarithm of the mutational frequency of the corresponding gene as described in Quesada *et al*. (2011) and Wang *et al*. (2011) ([24,25], respectively). Frequencies have been corrected for gene size and codon composition as reported in Quesada *et al.* Genes in red are mutated preferentially in *IGHV*-unmutated cases, genes in blue are mutated in *IGHV*-mutated cases and genes in purple are mutated in both.

**Figure 2:**
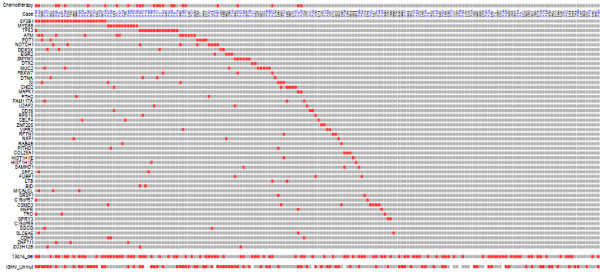
**Distribution of frequent mutations in CLL.** Red boxes indicate which patients carry somatic mutations in any of the 50 most frequently mutated genes in CLL from two whole-exome studies: Quesada *et al.* (2011) (case ID in black) and Wang *et al*. (2011) (case ID in blue) ([24,25], respectively). Red boxes also indicate whether the patient was treated before sample collection (Chemotherapy), whether deletion of 13q14 was detected (*13q14_del*), and whether the disease was classified as *IGHV*-unmutated (*IGHV-*Unmut).

This study was followed by two simultaneous whole-exome analyses of larger cohorts of CLL patients, one in the context of the ICGC [24] and one from the Broad Institute [25] (Figure 1). In addition to confirming the recurrent deletion in *NOTCH1*, both studies uncovered protein-altering somatic mutations that recurrently target multiple genes with varying frequencies (Figure 2). Thus, the gene encoding SF3B1 accumulates somatic mutations in about 10% of the CLL cases. In spite of its known role in the housekeeping process of splicing, this component of the U2 snRNP spliceosome is the target of antitumor drugs [26,27], which suggests that *SF3B1* is a *bona fide* target of driving mutations in CLL. Moreover, several reports have uncovered frequent somatic mutations affecting this gene in myelodysplasia [28,29] and other malignancies [30], including solid tumors [31-33]. In virtually all cases, mutations affect the C-terminal HEAT-repeat domain of SF3B1, and seem to cluster in a spatial region of its structure, which suggests that they disrupt the binding of the protein to some co-factor, which, in turn, might decrease the splicing fidelity in specific genes [24]. Consistent with this, and with the essential function of splicing in eukaryotic biology, mutations in *SF3B1* do not lead to widespread changes in the splicing patterns of tumor cells, as assessed with RNA-Seq [24,32,34,35]. It is worth noting that SF3B1 also plays a role in polycomb-mediated repression of *Hox* genes and, therefore, its role in tumor development might be independent of RNA splicing [36]. Since the therapeutic targeting of this protein might provide novel strategies for the treatment of a large number of CLL patients with a disease resistant to existing drugs [37], further studies aimed at determining the exact mechanism that connects SF3B1 mutation to tumor development are warranted.

As expected, there is a significant overlap between the genes that are frequently mutated in these whole-exome studies of CLL, even though some differences exist, mainly due to the low number of cases and the low frequency of recurrently mutated genes [24,25,38]. The highest deviation occurs with mutations affecting *TP53*, with one mutation out of 105 cases in Quesada *et al*. and 15 mutations out of 91 cases in Wang *et al*. (Figure 2). This discrepancy is likely due to the lower risk of CLL progression in the population-based, untreated cohort of the first study. In fact, *TP53* mutation is much more frequent in patients who have received chemotherapy prior to sample extraction. Consistent with this, *TP53* mutation and related alterations have been associated with disease progression and chemo-refractoriness in CLL [39,40]. Nevertheless, these differences do not affect the clinical consequences of *SF3B1* mutation [41,42].

## Epigenomic alterations in chronic lymphocytic leukemia

Recently, these genomic studies have been complemented with the first large-scale analysis of the epigenomic alterations in CLL [43]. In this work, a combination of whole-genome bisulfite sequencing and high-density microarrays was employed to characterize the methylomes of 139 CLL patients and several B-cell subpopulations. The results suggest widespread epigenomic reprogramming events during the development of this disease. Surprisingly, the main feature found in this study was hypomethylation inside the body of genes, which associates with the clinical characteristics of each sample. The inspection of this methylation signature suggests that, in addition to *IGHV*-mutated and *IGHV*-unmutated patients, there is a third clinical group of CLL patients with intermediate phenotypic characteristics. Additionally, the comparison of the methylation patterns between B-cell populations led to the conclusion that malignant *IGHV*-unmutated B-cells are related to pre-germinal center B cells, whereas malignant *IGHV*-mutated B-cells probably stem from germinal center-experienced B-cells [43]. Finally, CLL samples with mutations in *NOTCH1* and *SF3B1* show a distinct DNA methylation pattern, which suggests interplay between the most frequent genomic events and the epigenetic reprogramming associated with this neoplasia.

## The long tail problem in cancer genomics

As one considers less frequently mutated genes in CLL, the so-called problem of the long tail becomes apparent [44]. This problem arises when even the highest mutational frequencies in driver genes are low. As a consequence, there is a ‘long tail’ of extremely low-frequency driver mutations, which hinders the compilation of a complete catalogue that recapitulates the key genomic events for every patient. Thus, even when the 50 most frequently mutated genes are considered, a large number of patients show no mutation in any of them (Figure 2). Notably, deletions in 13q14 are more frequent, but this event by itself has mild clinical consequences [45]. This long tail problem challenges the search for drivers of CLL progression and, therefore, the search for novel guided therapeutic interventions, by statistical analysis of mutational frequency alone. To overcome this obstacle, several factors may be considered. First, normal whole-genome and whole-exome experiments are not sensitive enough to detect sub-clonal populations in newly-diagnosed patients. Therefore, some driver mutations may be invisible to these techniques until the sub-clones develop [46,47] or are selected for because of their resistance to chemotherapy [48]. This feature of CLL progression may be of paramount importance for the early prognosis and treatment choice in this disease. Thus, Landau *et al*. have shown in a recent manuscript that the presence of certain somatic mutations even in a small population of the tumor cells accurately predicts a high probability of relapse after treatment [49]. In fact, most of the driver mutations identified previously occur at sub-clonal levels at diagnosis. Only mutations affecting *MYD88*, chromosome 12 trisomy and del(13q) were classified as early clonal events. The authors also showed that clonal evolution is strongly associated with chemotherapy, with sub-clones containing mutations in genes like *SF3B1* and *TP53* expanding over time [49]. If this information is known at diagnosis, clinicians may choose to target those drivers early to prevent relapse, a strategy which we have called anticipation-based chemotherapy (ABC) [50].

In addition, low-frequency driver mutations in CLL may appear at higher frequencies in other malignancies. For instance, even though mutations affecting *BRAF* are infrequent in CLL, they target the same residues as those in diffuse large B-cell lymphoma, and are clustered with those found in hairy-cell leukemia [24,51]. Similar reasons suggest that mutations in *CCND2* may drive the development of CLL in a small percentage of cases [18]. These examples illustrate the putative relevance of infrequent mutations for the pathogenesis of CLL. An additional strategy for the characterization of the mutational landscape of CLL stems from the fact that driver mutations are expected to impact the functional residues of the encoded gene products. Therefore, after predicting the functional impact of the somatic variants, infrequently mutated genes may be classified as putative drivers if they accumulate more damaging mutations than expected by chance [52,53]. A similar method groups mutations that affect a given domain, even if they do not target the same gene [54].

Finally, algorithms to predict driving mutations may benefit from biochemical information by grouping together genes that belong to the same biological pathways. Usually, these genes are mutated in a mutually exclusive way. This approach may yield important clues about the biochemical determinants of CLL progression, although the low frequencies involved preclude the assessment of mutual exclusion in most cases. Nevertheless, the alteration of several pathways, including inflammatory response, pattern recognition, DNA damage, RNA splicing and telomere maintenance, has been postulated as a crucial step in this process [24,25,55]. Moreover, extensive mining of whole-exome and RNA-Seq data showed that up to one-third of the examined patients suffer somatic mutations affecting at least one component of the RNA maturation pathway, including splicing, exon-junction complex, regulation of alternative splicing and transport [34]. However, the pattern of mutations suggests that most of the grouped mutations are probably passengers of CLL development. The exception would be RNA transport, in which three genes (*NXF1*, *XPO1* and *DDX3X*) are recurrently mutated. Notably, all of the cases with mutations affecting this pathway were classified as *IGHV*-unmutated, which suggests that said mutations may be involved in the development of this aggressive form of the disease.

All these factors considered, there is a pressing need for direct biochemical experiments that confirm and extend these conclusions. Recent works have tried to recapitulate the biological consequences of some of the described mutations. These studies are highlighting new pathways in CLL development. For instance, frequent mutations in *POT1*, a gene whose product binds telomeric DNA and the shelterin complex, seem to affect telomere maintenance in patient cells [56]. Also, metabolic reprogramming might be involved in this malignant transformation through mutations affecting SI, a type-II transmembrane enzyme involved in carbohydrate metabolism [57]. These experiments must be extended with the development and use of animal models of CLL to answer some of the most challenging open questions and investigate new potential pharmaceutical interventions [58-60].

## Clinical perspectives

The newly acquired data depicting the genomic landscape of CLL have immediately suggested novel and interesting paradigms for the clinical management of this frequent form of leukemia. For instance, RNA-splicing inhibitors may provide a previously unsuspected strategy for the treatment of this neoplasia [61]. Also, somatic mutations affecting *NOTCH1*, *SF3B1* and *TP53* are improving our ability to predict the outcome of the disease from early stages [45,62]. Several studies have already been carried out to clinically characterize the consequences of mutations affecting these genes on the prognosis of CLL. Thus, the German CLL Study Group has sequenced samples from a large cohort of 1,124 newly diagnosed patients to ascertain the impact of *SF3B1* mutations on prognostic markers [63], whereas the CLL8 trial was interrogated for *TP53*, *NOTCH1* and *SF3B1* mutations and their relationship with treatment outcome [64]. These studies have confirmed that *SF3B1* mutation correlates with adverse prognostic features, with independent prognostic value on time to treatment, and shown that mutations in *NOTCH1* and *TP53*, but not *SF3B1*, associate with refractory disease. Importantly, a randomized, prospective clinical study in the context of the British LRF CLL4 trial has independently demonstrated the prognostic value of *NOTCH1* and *SF3B1* mutations, which single out a new group of patients with poor outcome after treatment with chemotherapy [65]. However, given the long tail of infrequent mutations in CLL, the future of patient care may depend on our ability to integrate the genomic and epigenomic data into a few common biochemical pathways. If this proves feasible, intervention on those pathways may lead to broad-spectrum treatments which improve OS and PFS for most patients. On the other hand, it seems at this point that CLL development is driven independently by changes in multiple, unrelated biological pathways. This would force us to integrate the genomic information into a personalized medicine framework to make sure that a large percentage of patients receive adequate care. In this scenario, individual genome sequencing would become a routine clinical test for precise diagnosis, prognosis and targeted therapeutic approaches. Obviously, this would require solving a wealth of shortcomings, ranging from our limited knowledge of the influence of mutations on treatment response to the necessity to take into account sub-clonal events and microenvironmental interactions which may shape CLL progression [66,67]. As genome-sequencing, information-processing and data-storing costs keep decreasing, this scenario becomes increasingly realistic. Therefore, it seems likely that information on patient response to treatment in relationship to the genomic events that drive each CLL case will keep growing to the point where genomic and epigenomic information will become an important factor in the clinical management of this disease.

## Conclusions

For the first time, we have a comprehensive view of the genomic landscape of CLL. This provides new biochemical targets for therapeutic intervention and improved staging procedures. While the resulting model needs to be experimentally validated, at this point it seems unlikely that a single strategy can be employed for the clinical management of a large percentage of patients. Therefore, it may be necessary to integrate personalized genomic information at diagnosis into the clinical decision-making process.

## Abbreviations

ABC: Anticipation-based chemotherapy; CLL: Chronic lymphocytic leukemia; ICGC: International Cancer Genome Consortium; NGS: Next-generation sequencing; OS: Overall survival; PFS: Progression-free survival; SHM: Somatic hypermutation; TCGA: The Cancer Genome Atlas.

## Competing interests

The authors declare no competing interests.

## Authors’ contributions

VQ and CL-O wrote the first draft of the manuscript with contributions from AJR and DR and critical reviews by XSP and EC. All authors read and approved the final manuscript.

## Authors’ information

The authors participate in the Spanish CLL project of the ICGC, supervised by CL-O and EC. The rest of the authors have been involved in detection and biochemical characterization of somatic mutations in CLL.

## Pre-publication history

The pre-publication history for this paper can be accessed here:

http://www.biomedcentral.com/1741-7015/11/124/prepub
